# Alteration of gene expression by exposure to a magnetic field at 23 kHz is not detected in astroglia cells

**DOI:** 10.1093/jrr/rrt063

**Published:** 2013-05-30

**Authors:** Tomonori Sakurai, Eijiro Narita, Naoki Shinohara, Junji Miyakoshi

**Affiliations:** Laboratory of Applied Radio Engineering for Humanosphere, Research Institute for Sustainable Humanosphere, Kyoto University, Gokasho, Uji, Kyoto, 611-0011, Japan

**Keywords:** induction heating (IH) cooktop, intermediate-frequency (IF) magnetic field, cDNA microarray, human-derived cells, International Commission on Non-Ionizing Radiation Protection (ICNIRP) guidelines

## Abstract

The increasing use of induction heating (IH) cooktops has roused public concern in Japan and Europe regarding potential health effects. The purpose of this study was to evaluate the effects of exposure to a magnetic field at 23 kHz (which is the maximum output power frequency of most IH cooktops) on gene expression in a human-fetus-derived astroglia cell line, SVGp12. The cells were exposed to the magnetic field at 2 mT_rms_ [which is approximately 74 times higher than the reference level in the most recent International Commission on Non-Ionizing Radiation Protection (ICNIRP) guidelines], for 2, 4 and 6 h, using a previously reported exposure system. Gene expression was evaluated using an Agilent cDNA microarray. We did not detect any significant effects of the magnetic field on the gene expression profile. On the contrary, heat treatment at 43°C for 2 h used as a positive control significantly affected gene expression, including inducing heat shock proteins, which indicated that our protocol for microarray analysis was appropriate. From these results, we conclude that exposure of human-fetus-derived astroglia cells to an intermediate-frequency magnetic field at 23 kHz and 2 mT_rms_ for up to 6 h does not induce detectable alteration of gene expression.

## INTRODUCTION

In Japan and Europe, gas and electric cookers have increasingly been replaced by induction heating (IH) cooktops in recent years. IH cooktops generate magnetic fields at 20 to 100 kHz, which belong to the intermediate-frequency (IF) range (300 Hz –10 MHz), from heating coils. These magnetic fields induce currents in metal cooking pans to heat the pans for cooking. In most IH cooktops, a magnetic field at 23 kHz is used as the maximum output power frequency, and some of the magnetic field leaks out. This has led to public concern in Japan regarding the potential health effects of IF magnetic fields leaked from IH cooktops.

Most previous epidemiological [1–4] and *in vivo* studies [5–7] on IF magnetic fields have focused on the effects on women of working with video-display terminals. In contrast, there have been few similar studies of IH cooktops, although several *in vitro* genotoxicity studies have been reported [8–10]. However, *in vitro* studies of the effects of IH cooktops on gene expression are still lacking, despite the crucial role of gene expression in maintenance of living systems.

In the past decade, powerful high-throughput transcriptomic and proteomic screening techniques have been used to study the effects of exposure to electromagnetic fields [11, 12]. Recently, we used a whole genome cDNA microarray technique [13] to investigate the effects of a 23 kHz magnetic field at 100 µT_rms_ for 2, 4 and 6 h in human-fetus-derived astroglia cells. There were no detectable effects of the IF magnetic fields at 23 kHz on the gene-expression profile. One hundred μT_rms_ is approximately 16 times higher than the reference level for general public exposure for magnetic fields of 3 kHz–10 MHz in the 1998 International Commission on Non-ionizing Radiation Protection (ICNIRP) guidelines [14].

However, in the recently updated ICNIRP guidelines, the reference levels for magnetic fields of 1–100 kHz have been revised, and the level for general public exposure to magnetic fields of 3 kHz – 10 MHz has been raised to 27 µT_rms_, and for occupational exposure has been raised to 100 µT_rms_ [15]. This revision raises a question about our previous study regarding the evaluated magnetic flux density. The larger the magnetic flux density, the larger the internal electric fields induced [14, 15]. The obvious biological effects were reported for larger internal electric fields than the threshold [14, 15]. Therefore, investigation of even larger magnetic flux density than the reference levels will provide important information for determining guidelines. The level of 100 μT_rms_, which we previously investigated [13], is insufficient since the multiple of 100 µT_rms_ to reference level for public exposure dropped to only ∼ 3.7 and equal for occupational exposure [15]. In addition, the World Health Organization (WHO) has recommended the carrying out of high-quality research to assess biological effects in the IF range, because there is limited experimental evidence to support the new reference level [16].[Fig RRT063F1]

**Fig. 1. RRT063F1:**
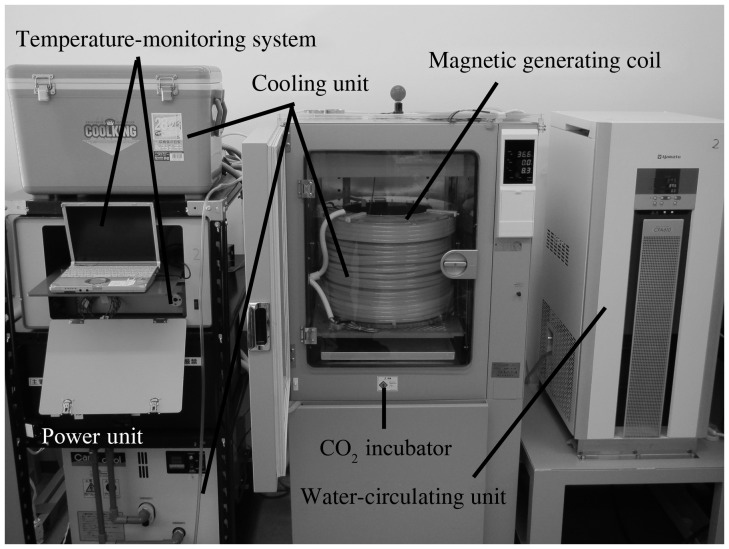
Picture of the experimental system used for exposure to intermediate-frequency magnetic fields. The system consists of a magnetic generating coil, a power unit, a cooling unit, a temperature-monitoring system, a CO_2_ incubator, and a water-circulating unit.

In this study, in response to the gaps in knowledge that have emerged since our previous work [13], we planned to investigate the effects of a magnetic field of 23 kHz and 2 mT_rms_, (i.e. ∼ 74 times higher than the reference level in the new ICNIRP guidelines), on gene expression in a human-fetus-derived astroglia cell line, SVGp12, using microarray analysis of cDNA gene expression. When a pregnant woman uses an IH cooktop, she is generally exposed to an IF field in the abdominal region, and the fetus in her abdominal region would also be exposed. Therefore, fetus-brain-derived cells were suitable for evaluating IF effects. In addition, the possibility of differences between species indicates the necessity of undertaking studies using not only animal but also human-derived cells; the possibility of differences in gene expression between tumor and normal cells supports the necessity of studies using normal cells. However, human-brain-derived normal cells are hard to obtain and grow. To solve this problem, we used SVGp12 cells.

## MATERIALS AND METHODS

### Magnetic field generation system

We used a previously reported [17] custom-built IF magnetic field generation system with a change of the coil current (Fig. 1). Briefly, this system consists of a custom-built magnetic generating coil, a power unit (KZ-MS32A; Panasonic, Osaka, Japan), a cooling unit (Carry Cool LPA3; Orion Machinery Co., Nagano, Japan), a temperature-monitoring system comprising an optical fiber thermometer (UM14; FISO Technology, Quebec, Canada) with a data acquisition/switch unit (34970A; Agilent Technologies, Palo Alto, CA), an incubator (modified BNR-110; Espec Corp., Osaka, Japan), and a water-circulating unit (modified Neo Cool Circulator CFA610; Yamato Scientific Co., Tokyo, Japan). The power unit was regulated to supply a current to the coil of 6.1 A_rms_ at 23 kHz to generate an IF magnetic field of 2 mT_rms_ from the coil. The magnetic field had a sinusoidal wave-form with a vertical and uniaxial orientation. The magnetic flux density in the exposure space was measured using an ELT-400 tester (Narda STS, Pfullingen, Germany) with a 3 cm^2^ probe (Table 1). The temperature of the medium in the culture dishes was monitored throughout the experiment and maintained at 37.0 ± 0.5°C. Sham exposure was performed simultaneously with IF exposure.

**Table 1.** Magnetic flux density for each Petri dish

Dish positions	Exposure system A		Exposure system B	
	Center (mT_rms_)	Periphery (mT_rms_)	Center (mT_rms_)	Periphery (mT_rms_)
1st layer	1.85	1.89	1.85	1.88
2nd layer	1.94	1.98	1.92	1.97
3rd layer	1.94	1.99	1.93	1.97
4th layer	1.85	1.90	1.85	1.89
Variation	4.6%		3.9%	

### Microarray experiment

The human-fetus-derived astroglia cell line, SVGp12 (American Type Culture Collection, Manassas, VA), was cultured with Eagle's minimum essential medium (Nikken Bio Medical Laboratory, Kyoto, Japan) supplemented with 10% fetal bovine serum (BioWest, Miami, FL), 0.1 mM non-essential amino acids, and 1 mM sodium pyruvate. Cells were seeded at a density of 1 × 10^6^ cells/dish in 100-mm diameter dishes and cultured for 24 h. Immediately after exposure to the IF magnetic field for 2, 4 or 6 h, total RNA was extracted using an RNeasy Mini kit (Qiagen, Hilden, Germany). The concentration and purity of extracted RNA was measured using an UV-visible spectrometer (NanoDrop 1000; Thermo Fisher Scientific, Waltham, MA) by absorption at wavelengths of 260 and 280 nm. RNA samples with a 260/280 nm absorption ratio >1.8 were used in a subsequent microarray analysis.

An amplification reaction with simultaneous introduction of Cy3 to the amplified complementary RNA (cRNA) was performed using a Quick Amp Labeling Kit for One-Color (Agilent Technologies), starting with 500 ng of total RNA. After purification of the Cy3-labeled cRNA, the Cy3 dye incorporation efficiency and concentration of cRNA were evaluated using a NanoDrop 1000 spectrophotometer at 550 nm and 260 nm, respectively. After fragmentation of the dye-labeled cRNA at 60°C for 30 min, samples were hybridized on a DNA chip (Whole Human Genome (4 × 44K); Agilent Technologies, 43 376 gene spots) at 65°C for 17 h in a hybridization oven (Agilent Technologies). Three independent experiments were performed under each experimental condition.

### Heat treatment as a positive control

To validate the microarray analysis protocol for IF exposure, alteration of gene expression was examined in cells treated at 43°C for 2 h. The cells were seeded at a density of 1 × 10^6^ cells/dish in 100-mm diameter dishes, cultured for 24 h, and then incubated at 43°C for 2 h in a water bath. Total RNA was extracted immediately after the heat treatment. The subsequent microarray procedure was the same as that for IF exposure. Three independent experiments were performed.

### Data analysis

Hybridized DNA chip slides were scanned using an Agilent Scanner (Agilent Technologies) with Feature Extraction Software. Fluorescence intensity data were imported to GeneSpring GX version 11.0.2 (Agilent Technologies) with the 75th percentile scaling normalization. Before analysis, four data-filtration steps were conducted to exclude low-quality data to guarantee the accuracy of the statistical analysis. In the first step, spots with lower intensities than the threshold, which was determined based on the intensities of the Agilent RNA Spike-Mix, were filtered out to exclude weaker spots than background noise. In the second step, spots with saturated intensities and near-background intensities were filtered out using the ‘flag’ function of the Feature Extraction Software. In the third step, spots with large variance among the three repeated experiments (coefficient value >50%) were filtered out. The final filtration step was conducted based on the fold increase. Statistical analysis of the genes remaining after the four filtration steps was performed with a *t*-test corrected using the Benjamini and Hochberg false discovery rate.

## RESULTS

### Number of remaining gene spots after filtration steps

Since the accuracy of the analysis depends on the quality of gene spots subjected to analysis, we conducted data filtration before analysis. After filtration, the number of gene spots in IF exposure for 2, 4 and 6 h, and heat treatment were 21 254, 20 854, 23 056, and 22 707, respectively, indicating similar values for the four experimental conditions. Not all genes mounted on the cDNA chip were expressed in SVGp12 cells because the gene spots were selected for evaluation in multiple human-derived cell lines. Therefore, the results obtained after filtration are reasonable.

### Differential gene expression in cells exposed to an IF magnetic field

The number of gene spots judged to be altered in expression by exposure to an IF magnetic field are shown as the fold increase in Table 2. No genes showed a significant change in IF-exposed cells. The number of gene spots altered by exposure to an IF magnetic field (without statistical analysis) is shown in parentheses in Table 2. Based on a cutoff value of 2-fold, only a few gene spots were altered in each exposure condition. Based on a 1.5-fold cutoff, 0.06–0.11% of gene spots were altered. Fold changes of 2.0 [18–20] or 1.5 [21, 22] are common cutoff values in microarray experiments. Therefore, we concluded IF exposure causes very few changes in gene expression.

**Table 2.** The number of gene spots judged to be ‘altered’ by exposure to IF magnetic fields after statistical analysis, and without statistical analysis

Exposure Duration (h)	Regulation	Fold change			
		>3.0	>2.0	>1.5	>1.1
2	Up	0 (0)^a^	0 (2)	0 (33)	0 (1 248)
	Down	0 (0)	0 (1)	0 (14)	0 (960)
4	Up	0 (0)	0 (1)	0 (22)	0 (1 204)
	Down	0 (0)	0 (1)	0 (14)	0 (1 575)
6	Up	0 (0)	0 (2)	0 (15)	0 (827)
	Down	0 (0)	0 (1)	0 (10)	0 (690)

^a^The number in parenthesis represents the result without statistical analysis.

### Differential gene expression in cells treated at 43°C for 2 h

To validate our protocol, we conducted microarray analysis of cells after heat treatment at 43°C for 2 h as a positive control. The experimental procedure after RNA extraction and data processing were performed as for the IF-exposed cells. A large number of gene spots showed a significant change compared with the IF-exposed groups (Table 3), including altered expression of genes encoding heat shock proteins and stress response proteins: FBJ murine osteosarcoma viral oncogene homolog (NM_005252), 186-fold upregulated; FBJ murine osteosarcoma viral oncogene homolog B (NM_006732), 21-fold upregulated; heat shock 70 kDa protein 1A (NM_005345), 18-fold upregulated; crystallin, alpha B (NM_001885), 14-fold upregulated; DnaJ (Hsp40) homolog, subfamily B, member 1 (NM_006145), 13-fold upregulated.

**Table 3.** The number of gene spots judged to be ‘altered’ by heat treatment after statistical analysis, and without statistical analysis

	Fold Change			
Regulation	>3.0	>2.0	>1.5	>1.1
Up	54 (55)^a^	138 (168)	490 (699)	2 962 (9 015)
Down	19 (19)	226 (228)	1 129 (1 186)	2 774 (5 618)

^a^The number in parenthesis represents the result without statistical analysis.

## DISCUSSION

In this study, we evaluated the effects of exposure to 23 kHz magnetic fields at 2 mT_rms_ for 2, 4 and 6 h on gene expression using a DNA microarray for high-throughput transcriptomic screening. No effects of IF exposure on SVGp12 cells were detected and there was no significant alteration in gene expression in these cells after exposure (Table 2). The results (without statistical analysis) showed that a small number of genes was altered by IF exposure compared with heat treatment (Tables 2 and 3).

Based on the results of heat treatment (Table 3), we concluded that the experimental procedures and data processing in this study were conducted appropriately. Significant changes in gene expression were detected in the heat-treated cells, including changes in levels of heat shock protein 70; DnaJ (Hsp40) homolog, subfamily B, member 1; FBJ murine osteosarcoma viral oncogene homolog; FBJ murine osteosarcoma viral oncogene homolog B; and αB crystallin, all of which have been previously reported to be affected by heat treatment [23–26]. The consistency of our microarray results with previous gene expression reports indicates that the experimental procedures and data processing were performed appropriately in this study, and that the cells used were responsive to external toxicological stimulation. This excludes the possibility that the lack of detection of significant changes in gene expression in cells exposed to an IF magnetic field was due to the non-responsiveness of the cells used in the study.

The absorption of energy from electromagnetic fields at frequencies below about 100 kHz is normally negligible, and no measurable temperature rise occurs in the body, whereas exposure to electromagnetic fields at frequencies above 100 kHz leads to significant absorption of energy and related temperature increases [14]. Based on the profile of energy absorption from magnetic fields, and the results of this study conducted at under 37.0 ± 0.5°C, we conclude that exposure to a magnetic field at 23 kHz and 2 mT_rms_ for up to 6 h does not induce detectable alteration in gene expression in SVGp12 cells.

## CONCLUSION

In Japan, approximately 3 million households are now using IH cooktops. The increasing use has raised public concern regarding the potential health effects of IH cooktops. The findings in this study show that magnetic fields at 23 kHz do not have any detectable effects on gene expression in human-fetus-derived astroglia cells. We note that the experimental conditions in this study do not fully reflect actual human exposure to IH cooktops, because the waveforms and frequency spectrum of IH cooktops are more complicated, and humans are exposed to magnetic fields intermittently and with long-term and complex exposure. However, we found that exposure for 6 h to a magnetic field at the frequency of IH cooktops, and at ∼ 74 times higher magnetic flux density than the reference level in the updated ICNIRP guidelines, did not cause detectable alteration of gene expression. This provides a basis for improved understanding of the potential health effects of IH cooktops.
